# Conference Report: WORKSHOP ON NEW MEASUREMENT TECHNOLOGY FOR POLYMER PROCESSING Gaithersburg, MD December 3–4, 1990

**DOI:** 10.6028/jres.096.027

**Published:** 1991

**Authors:** Anthony J. Bur, Pawan Handa, Chris Grudzien

**Affiliations:** Polymers Division, National Institute of Standards and Technology, Gaithersburg, MD 20899; The Goodyear Tire & Rubber Co., Goodyear Research, 142 Goodyear Blvd., Akron, OH 44305; Dynisco Inc., Four Commercial St., Sharon, MA 02067

This report describes activity at a workshop on New Measurement Technology for Polymer Processing which was held at the National Institute of Standards and Technology on December 3–4, 1990. The workshop was attended by 19 industry scientists and engineers from polymer processing and instrumentation manufacturing companies. The objective was to seek industry responses to the question: what in-line or on-line, real-time measurements do you need to make during polymer processing but are unable to do so because the measurement technology does not exist? Processors identified their measurement problems and needs by describing various processing operations. Tire and rubber manufacturing, thermoplastic compounding, injection molding, film processing, and reactive processing were described with respect to related measurement problems. Workshop discussions yielded a consensus of the following measurement needs: (a) in-line rheological measurements; (b) improved in-line temperature measurements; and, (c) in-line and on-line measurements of polymer morphology. An ongoing NIST measurement development program, which focuses on temperature and rheological measurements, was described. The NIST program is based on optical and fluorescence measurement techniques which have been used both for laboratory experiments and for instrumenting process machinery. The workshop is being used as a basis for developing NIST/industry collaboration on measurement technology development.

## 1. Introduction

The National Institute of Standards and Technology (NIST) was host to industry scientists and engineers at two recent workshops on technical problems associated with polymer processing. These workshops, held at NIST in September 1988 and December 1990, focused on obstacles and challenges which prevent polymer processors from utilizing the full potential of microprocessor based control to effect process control and thereby improve product quality and processing productivity.

The **1988** workshop had rather broad objectives:

- To identify processing methods that would benefit from automation and improved understanding of the process;
- To isolate important generic problems that limit the automation and productivity and impede the processing of high quality polymer materials;
- To identify technical and scientific barriers that must be overcome in order to solve processing problems;
- To identify concepts, measurement techniques, and other tools that can be applied to these problems; and
- To define the respective roles of NIST and the industrial sector in solving these problems and to establish communication between NIST and the U.S. polymer processing community.

A consensus regarding the technical barriers which face the processing industry unfolded from the discussions and presentations at the **1988** workshop. They are:

- On-line and in-line, real-time measurements;
- Process models;
- Fundamental theoretical understanding of the physics, rheology, and chemistry of polymer processing;
- Rheological understanding and structure/property relationships of new materials; and
- Materials databases.

Improvements in all of the above areas are needed in order to effectively employ program and microprocessor control based on artificial intelligence. It was agreed that the first two items, on-line measurements and process models, were the areas of most immediate need. Within the context of process control, the measurement problem was stated as follows: (a) The control of a process parameter first requires its measurement; and, (b) The importance of a process parameter will be identified by its expression in a mathematical process model. Improved and new measurement technology is of primary importance because process control cannot be achieved without it and because measurements are needed to test process models. A detailed report of the 1988 workshop is available from NIST [1].

The **1990** workshop on *New Measurement Technology for Polymer Processing* was held at NIST on December 3–4, 1990. This workshop had a single focus:

- **On-line and in-line measurement problems associated with polymer processing.**

In attendance were 19 representatives from the polymer processing and instrument manufacturing industries. The theme of the workshop centered around the question: what in-line or on-line measurements do processors wish to make but are unable to do so because the technology does not exist? The answer to this question emerged from presentations by workshop participants, from open discussions, and from responses to a questionnaire. Measurement problems were highlighted during industry presentations about injection molding, compounding, extrusion, reactive processing and film processing. Also, information was acquired by distributing a questionnaire through which we posed questions to processors concerning process problems and real-time measurement needs. Three areas of measurement needs evolved: *in-line rheological measurements, improved in-line temperature measurements*, and *in-line and on-line morphology measurements.* Of somewhat less importance was the need for chemical analysis during reactive processing.

The 1990 workshop activity was scheduled in four sessions:

- Measurement Needs of the Polymer Processing Industry;
- The NIST Polymer Processing Measurement Program;
- Measurement Technologies from the Instrument Manufacturer’s Perspective;
- Future NIST/Industry Collaboration on Measurement Development Programs.

## 2. Measurement Needs, an Industry Perspective

The rationale for performing accurate and reliable on-line and in-line processing measurements was stated in a number of different ways by industry processors. For example, measurements are needed in order to: save money on post processing characterization, maintain and improve product quality, reduce off-specification products, control process, control materials properties of product, reduce time for off line post characterization, improve yield and cycle time, maintain product uniformity, control melt flows, monitor process and correct deviations from specification quickly, control reactive processing, and continuously improve product. All of these reasons can be condensed into one phrase: *product quality and process productivity.* All workshop participants recognized that improved process measurement and control are the principal means for achieving their quality and productivity objectives.

Industry scientists described a number of processing methods for which outstanding measurement problems exist: tire and rubber processing, polymer compounding, injection molding, film processing, and reactive processing. Several common themes of measurement needs emerged from these talks, namely, measurements of Theological and flow properties; temperature and temperature gradients; morphological parameters such as composition, degree of mix and domain size; and chemical analysis of reactive processing. This list is essentially the same as that which developed from the 1988 workshop. It is recognized that new measurement technology development is necessary because existing capabilities do not fulfill current requirements. Some participants stated needs for good processing models, in agreement with comments made at the 1988 workshop. Although processing models are of large importance to the processing industry, the primary focus of the 1990 workshop was on-line and in-line processing measurements.

The most frequently mentioned measurement need was for in-line, real-time rheological measurements. An important distinction was made between in-line and on-line measurements of rheological parameters. On-line refers to measurements made on a sub-stream of material diverted from the main process line, whereas in-line refers to measurements made on the material while it is in the main process line. The consensus communicated by the workshop participants was that in-line measurements are the more important in terms of monitoring and controlling the process. For rheological measurements, *on-line* observations will yield materials data for the polymer being processed, but do not describe the manner in which the polymer expresses itself Theologically in response to the processing stresses imposed on it.

Several processing operations, film extrusion, rubber manufacturing and extrusion, and injection molding, were presented as processing operations which would benefit from in-line rheological measurements. For film extrusion, uniform thickness and mechanical properties of the final product depend on the volume throughput rate, temperature, and viscosity of the resin as it moves through the exit die. Changes in resin viscosity during this operation influence the volume flow rate so that the pull-off rate must be adjusted accordingly. In the case of tire and rubber manufacturing, several steps in the process would benefit from a real time viscosity and viscoelastic measurements. Knowledge of viscoelastic properties is a prominent requirement during mixing of rubber elastomer with fillers and during extrusion and calendering of the sheeted gum product. During injection molding, high volume flow rates are accompanied by shear rates which are normally not available with standard laboratory equipment. Thus, the viscosities expressed by the flowing resin are not known from an established calibration curve but are assumed from an extrapolation of data from lower shear rates. The direct, in-line measurement of viscosity, i.e., the ratio of shear stress to shear rate, will permit a test of the extrapolation accuracy and provide a method to detect shear degradation. Volume flow rate and its relationship to viscosity are fundamental process parameters which govern continuous processing of films and extrusion of rubber. Maintaining an optimum balance between temperature, volume flow rate, viscosity, shear stresses and shear rate is a primary objective. During reactive processing viscosity is a specifying materials property by which the chemical reaction or polymerization is monitored.

An extended discussion about the measurement and control of temperature and temperature gradients transpired. Temperature not only determines the rheology of the processed material but also polymer degradation and reaction kinetics during reactive processing. The measurement of temperature gradients, particularly during molding operations, is also required. The main point of concern is that temperature measurements need to reveal the temperature of the material itself without influence from temperature conditions of the machine. In order to avoid machine influences, probes are often inserted into the flow stream which can cause breakage. Mold cavity temperatures are particularly difficult to measure because of the large thermal mass of the mold which is held at a low temperature by a circulating coolant. During batch mixing, temperature measurement and control of the actual mix compound is desirable in order to avoid hot spots due to high shear loading and to minimize the energy expended on the mixing process. Processors related their experience dealing with all types of temperature sensors, thermocouples, semiconductor devices, resistive elements, and infrared radiometry. The consensus of the processing community was that there are many manufacturing situations where the existing technology is inadequate because a precise and accurate temperature of the polymer resin is not known.

The current emphasis on reactive processing, compounding, alloying and blending has created requirements for on-line and in-line morphological measurements. In particular, processors expressed a need to know molecular weight, molecular weight distribution, domain size in mixtures of immiscible resins, filler orientation, molecular orientation, and polymer microstructure. Classical morphological measurement techniques such as low angle x-ray scattering, neutron scattering, and electron and optical microscopy are not adaptable to processing operations. Advances in fundamental science and the development of new and creative measurement concepts are needed in order to meet this challenge.

Some discussion on pressure measurements and associated problems ensued, but the general view was that, while some problems exist here, they do not severely affect product quality and productivity. Some problems experienced with pressure measurements are slow response time compared to changes which occur in injection molding, fouling of the sensor by the resin so that true pressure is not transferred to the sensor, difficulty incorporating the sensor into the mold cavity, susceptibility to damage in the manufacturing environment.

## 3. The NIST Measurement Program

The direct transfer of information between NIST and industry at the 1988 and 1990 workshops provided a framework within which the NIST measurement program is defined. This program, based on the application of fluorescence spectrometry and optical measurement methods, addresses two industry measurement needs: in-line rheological and in-line temperature measurements. Optical measurements, particularly fluorescence spectrometry, offer new, unexplored opportunities for polymer processing. Although a large foundation of fundamental fluorescence and optical science exists, very few attempts to apply this knowledge to processing measurement problems have been carried out. Developing measurement instrumentation for the processing environment will require significant applied research and engineering. The ready availability of optical fibers, which can be placed in existing instrumentation ports on processing equipment, will facilitate the transfer of this technology.

Highlighted at the workshop were two successful applications to the processing environment, measurement of quality-of-mix of polymer resin with particulate filler, and monitoring the glass transition of polystyrene. For the quality-of-mix experiments, laboratory development was undertaken and a subsequent technology transfer to a processing facility was carried out. A twin screw extruder was instrumented with optical fibers and associated optics detection equipment in order to monitor the quality-of-mix, concentration, and residence time distribution of a resin/particulate mixture. The glass transition of polystyrene was monitored using a viscosity sensitive fluorescent dye which produced distinct fluorescence intensity changes associated with the onset of the glassy state. The measurement method can be applied to processing where a liquid to crystal or glass phase transition needs to be monitored.

For rheological measurements, a polymeric dye, which can engage in the entanglement network of the host matrix polymer, has been used [2]. Under the application of shear stress, the resulting molecular orientation was measured by observing the fluorescence anisotropy of the incorporated polymeric dye. A polymeric dye consisting of anthracene tagged to polybutadiene was synthesized and doped into a polybutadiene host. Simultaneous fluorescence, shear stress, and shear rate measurements, which were made using an optically instrumented cone and plate rheometer, showed that fluorescence anisotropy is dependent on shear stress. The application of this phenomenon to the processing line will permit the observation of shear stress from a measurement of fluorescence anisotropy.

For temperature measurements, temperature sensitive dyes such as molecular rotors or excimer producing dyes will be used. The fluorescence intensity of these dyes is dependent on temperature and microviscosity in their molecular neighborhood. A molecular probe will reflect the temperature of the host polymer resin without influence from the temperature of the processing machine. An additional advantage of this temperature measurement is its fast response time, typically 10^−4^ s. The inherent limitation of the fluorescence response time is on the order of 10^−8^ s. Two types of temperature sensitive fluorescent molecules will be used:

- Molecular rotor dyes [3, 4];
- Excimer producing dyes [5].

The measurement of temperature using these dyes will rely on a calibration function which is obtained for the particular polymer system being monitored. It is not expected that a universal calibration curve will emerge.

The Theological and temperature measurements programs form the core of the ongoing NIST development work.

## 4. Instrument Manufacturer’s Perspective

Representatives from the instrument manufacturing community presented an assessment of morphology and mixing measurements, infrared analytical measurements, and temperature and pressure measurements. Generally, speakers at this session of the workshop described in detail the measurement capabilities of instruments from their companies. In particular, the presentations on morphology and mixing, infrared measurements, and infrared radiometric temperature measurements were company and instrument specific and will not be described in detail here. The reader is referred to companies manufacturing these instruments for additional information.

Pressure measurements and pressure transducers for polymer processing were described in general, generic terms. The different types of pressure transducers and their pros and cons were evaluated. In most cases, the transducer consists of a flexing diaphragm which, under pressure, imposes a load on a strain sensitive electrical component such as a resistive element, a semiconducting element or a capacitor. Pressure ranges and sensitivities are controlled by varying the rigidity of the diaphragm and the sensitivity of the electrical component. Temperature compensation is usually required and is incorporated in the electronic package. High temperature applications require special designs such as a mercury filled transducer in which the electronics are separated from the high temperatures at the sensing end. Dynamic pressure transducers based on the response of a piezoelectric material are also available. The trend for future pressure transducer development is to use fiber optics with which the problem of electrical interference in the manufacturing plant is eliminated.

## 5. NIST/Industry Collaboration

The workshop succeeded in achieving direct communication between NIST and industry concerning existing measurement problems. Strong support was communicated for the NIST Theological and temperature measurements programs. In order to maintain and utilize these lines of communication, an invitation to industry to collaborate with NIST was issued, and positive responses were expressed by most of the processing industry representatives present. It was agreed that a plan for a joint program would be developed and presented to those who wish to participate in ongoing research based on optical measurement methods. The objective of this research is to develop in-line, real-time instrumentation for Theological and temperature observations.

## 6. Summary

This report describes the activity at a workshop on *New Measurement Technology for Polymer Processing* which was held at NIST on Dec. 3–4, 1990. In attendance were representatives from NIST and polymer processing and measurement instrumentation companies. The theme of the workshop centered around the question: what on-line or in-line, real-time measurements of processing parameters do you need to make but are unable to because the technology does not exist? Industry participants highlighted their measurement problems during discussions about injection molding, compounding, extrusion, reactive processing, and film processing. Three areas of measurement needs emerged from these discussions: in-line rheological measurements, improved in-line temperature measurements, and in-line and on-line morphology measurements.

The direct transfer of information between NIST and industry at this and another workshop held in 1988 has provided a framework within which the NIST measurement program is defined. This program, based on the application of fluorescence spectrometry and optical measurement methods, addresses two industry measurement needs: in-line rheological and in-line temperature measurements. Optical measurements, particularly fluorescence spectrometry, offer new, unexplored opportunities for polymer processing.

Strong support for the NIST measurement program was expressed by most of the processors attending the workshop. Based on this positive response, industry collaboration with NIST was invited. Information about this program may be obtained by contacting Anthony J. Bur at 301-975-6748.
